# The waiting time problem in a model hominin population

**DOI:** 10.1186/s12976-015-0016-z

**Published:** 2015-09-17

**Authors:** John Sanford, Wesley Brewer, Franzine Smith, John Baumgardner

**Affiliations:** Horticulture Dept., NYSAES, Cornell University, Geneva, NY 14456 USA; Fluid Physics International, P.O. 667606, Houston, TX 77266 USA; FMS Foundation, Inc., Waterloo, NY 13165 USA; Los Alamos National Lab (retired), Los Alamos, NM 87545 USA

**Keywords:** Biological information, Text strings, Nucleotide strings, Waiting time, Functional threshold, Evolution, Mutation density, Numerical simulation, Mendel’s Accountant

## Abstract

**Background:**

Functional information is normally communicated using specific, context-dependent strings of symbolic characters. This is true within the human realm (texts and computer programs), and also within the biological realm (nucleic acids and proteins). In biology, strings of nucleotides encode much of the information within living cells. How do such information-bearing nucleotide strings arise and become established?

**Methods:**

This paper uses comprehensive numerical simulation to understand what types of nucleotide strings can realistically be established via the mutation/selection process, given a reasonable timeframe. The program *Mendel’s Accountant* realistically simulates the mutation/selection process, and was modified so that a starting string of nucleotides could be specified, and a corresponding target string of nucleotides could be specified. We simulated a classic pre-human hominin population of at least 10,000 individuals, with a generation time of 20 years, and with very strong selection (50 % selective elimination). Random point mutations were generated within the starting string. Whenever an instance of the target string arose, all individuals carrying the target string were assigned a specified reproductive advantage. When natural selection had successfully amplified an instance of the target string to the point of fixation, the experiment was halted, and the waiting time statistics were tabulated. Using this methodology we tested the effect of mutation rate, string length, fitness benefit, and population size on waiting time to fixation.

**Results:**

Biologically realistic numerical simulations revealed that a population of this type required inordinately long waiting times to establish even the shortest nucleotide strings. To establish a string of two nucleotides required on average 84 million years. To establish a string of five nucleotides required on average 2 billion years. We found that waiting times were reduced by higher mutation rates, stronger fitness benefits, and larger population sizes. However, even using the most generous feasible parameters settings, the waiting time required to establish any specific nucleotide string within this type of population was consistently prohibitive.

**Conclusion:**

We show that the *waiting time problem* is a significant constraint on the macroevolution of the classic hominin population. Routine establishment of specific beneficial strings of two or more nucleotides becomes very problematic.

## Background

At the heart of all information systems, including biological information systems, are strings of symbolic characters. Within the human realm, communication is generally based either upon strings of sounds, strings of letters, or strings of binary bits. In the biological realm, much information is transmitted within the cell based upon long strings of either nucleotides or amino acids. The information within an RNA or protein string almost always traces back to DNA strings. Within DNA, a primary unit of functional information is the gene, which is a very long text string that can range in size from about 1000 to more than one million nucleotides long. No geneticist believes that these very long nucleotide strings could ever arise directly, from scratch, via the mutation/selection process. Rather, a new gene is thought to arise from a previously existing gene, with the mutation/selection process establishing mutations within the long text string that is already established and functional. If sets of mutations (or short sub-strings) can be established within the preexisting longer string, then a new gene might arise, having a new biological function. Since a typical human gene is roughly 50,000 nucleotides long, to form a new gene with a new function should typically require the establishment of multiple mutations (or multiple sub-strings) within a pre-existent gene.

In this paper we address the question, “How long does it take for the simplest biological text strings to arise and be fixed, within a hominin population?” Given the unique capabilities of humans, an evolving hominin population would need to establish a great deal of new information, leading to new functionalities. Since all information is based upon strings of symbolic characters, this suggests the need to establish a multitude of new nucleotide sub-strings. Chimp and human genomes are minimally 5 % divergent [1], representing about 150 million nucleotide differences. This substantial genomic difference implies many new sub-strings within the human genome. In this paper, we only examine the average waiting time needed to generate and fix a single DNA sub-string of minimal length (2–8 nucleotides). This sort of minimal genomic modification would alter only one (or a few) specific amino acids, or might conceivably result in one new specific protein fold.

Numerous scientists have previously examined the problem of waiting time. Haldane was the first to address this problem [2]. He realized that even after a highly selectable beneficial mutation arose within a given population, considerable time was required for the amplification of that new mutation – such that all individuals in the population inherited the new mutation (“genetic fixation”), thus driving the original allele to extinction. Haldane realized that for long-lived organisms such as *Homo sapiens*, waiting time for fixation appeared to be problematic (only allowing about 1000 beneficial mutations to be fixed within the evolving pre-human population during the span of 6 million years). This problem came to be known as *Haldane’s Dilemma*. Kimura [3] agreed with Haldane’s assessment of the problem, and this motivated him to develop his neutral model of evolution. He reasoned that since selective amplification is too slow to fix very many mutations, most genetic fixations must result from random genetic drift.

Although significant, the waiting time associated with Haldane’s Dilemma is eclipsed by a much more profound waiting time problem. Haldane assumed a continuous supply of high-impact beneficial mutations, and did not realize that such mutations occur very rarely. Waiting for such mutations to arise is a much more significant factor affecting waiting time than the time required to amplify a given beneficial mutation after it has arisen. It is now generally recognized that beneficial mutations are rare, and that high-impact beneficial mutations are extremely rare [4–9]. Because of this, waiting for just the right mutation to arise in just the right location can be a rate-limiting factor in terms of the long-term evolution of any relatively small population. When a population faces a specific evolutionary challenge, a specific fix is needed, and it must arise in a timely fashion. Positive selection cannot generally begin to resolve an evolutionary challenge until just the right mutation (or mutations) happens at just the right position (or positions). Selection for the required trait can only begin after the mutation (or mutations) result in a substantial (selectable) improvement in total biological functionality. In higher life forms where population sizes are modest, the mutation rate per nucleotide per generation is normally extremely low (about 10^−8^) [10–12] – so that waiting for a particular mutation to arise can require relatively deep time. The waiting time required for the generation and fixation of multiple specific mutations needed to combine to create a new function can require inordinately long waiting times.

Behe and Snoke [13] and Behe [14] have made the argument that when more than two specific mutations are required to create a specific new biological function, the waiting time problem can become prohibitive. To illustrate this point, Behe [14] used the historical case of natural selection for malarial resistance to the drug chloroquine, which required two mutually-dependent mutations. He used some rough calculations based upon malarial incidence data, and then extrapolated those numbers to a hypothetical human population. Arguably, there may be problems with that analysis, as well as the earlier analysis by Behe and Snoke [15–19]. It is beyond the scope of this paper to defend or refute those earlier studies. Regardless of the validity of the initial mathematical formulations of Behe and Snoke, the basic question they raised remains interesting, and their work has led to considerable additional research by other investigators (this paper; [15–26]).

Virtually all of the papers subsequent to the work of Behe and Snoke have confirmed that waiting times can be prohibitive – depending upon the exact circumstances. Some of the subsequent papers have been critical [15–17, 25]. Yet even those papers show that establishing just two specific co-dependent mutations within a hominin population of 10,000 can require waiting times that exceed 100 million years (see discussion). So there is little debate that waiting time can be a serious problem, and can be a limiting factor in macroevolution. The real question is; “How long must the required string of nucleotides be, before the waiting time becomes prohibitive?” As we will see, the answer to that question depends on the specific biological circumstances.

The major point of disagreement seems to involve the question of exactly how to formulate the waiting time problem. For example, is waiting time measured in terms of years to first instance of the string, or years until the string has arisen repeatedly – to the point where the string persists and goes to fixation? What constitutes a valid functional target string? Is a perfect nucleotide match required to create a functional string? Can the target string arise anywhere within the genome? Does selection begin when the string is complete (fully functional), or when it is only partially complete (limited function)? Are all of the “stepping-stone” mutations that lead to a selectable string neutral, or are some deleterious? How large a fitness benefit does the completed string provide?

The various researchers who have examined the issue of waiting time have approached the problem from different directions, using many different starting assumptions. This means that comparing their results can often be like comparing apples and oranges. It is unlikely that there is a universal way to characterize the waiting time problem, as each circumstance requires special treatment. For example, the size and generation time of a population has a large effect on waiting time. Some authors like Behe/Snoke, Lynch, Axe, and Gauger focus almost exclusively on mega-populations with very short generation times (i.e., microbes). Authors like Durrett and Schmidt focus on smaller populations with longer generations. In this paper we focus exclusively on the classic hominin population, as would give rise to modern man. It is in this type of population where the waiting time problem is most acute, and this is where our expertise in comprehensive numerical simulation is most useful. We do not claim that our findings involving hominin-type populations can necessarily be extended to microbial systems.

In this paper we take a fresh approach to the waiting time problem, using comprehensive numerical simulations that are designed to simulate the mutation/selection process in a biologically realistic manner – taking into account all relevant factors simultaneously. We use biologically realistic numerical simulations to analyze waiting times for the generation and fixation of specific strings of nucleotides of various lengths, given different mutation rates, given different selection pressures, and given different population sizes.

Our goal is *not* to support the formulations of Behe, or his supporters, or his critics – but simply to bring greater clarity to the waiting time issue. In order to cut through some of the confusion, we use the most easily understood formulation possible, so that both specialists and non-specialists can assess our findings with a reasonable level of understanding. For the purpose of clarity we made our formulation very specific, and we do not claim it is universally applicable. The specific features of our formulation are as follows: 1) we use biologically realistic numerical simulations to study the average waiting time required for the generation and the fixation of a specific string of nucleotides within a specific location of the genome; 2) we design the starting sequence so that it differs from the target sequence at every nucleotide site; 3) we specify that as the starting string begins to mutate, all the subsequent evolving strings are neutral in their fitness effect until an instance of the specified target string arises; 4) we specify that when an instance of the target string arises, a specific fitness benefit (a reproductive advantage) is assigned to each individual carrying that target string, with the target string being fully dominant; 5) the mutation/selection process is faithfully played out according to the standard understanding of the neo-Darwinian process. The only deviation from the classic Darwinian process in our simulation experiments is that once an instance of the target string arises, we protect it from further mutations (as we discuss in the methods section). This practical consideration is conservative, as it can only shorten waiting time.

We expect dialog regarding the details of our formulation. Some may say the string should be able to arise anywhere (genetic context does not matter). Some may say that the rewarded sequence should not have to match the specified target sequence exactly. Some may say that in a specified location, the string might already be there – perhaps lacking only a single new nucleotide. Some may say that each nucleotide in the string should be independently rewarded. In all these special circumstances the waiting time will certainly be shorter than this study indicates. But we are modeling, for the sake of clarity, a simple and well-defined model that is generic in its nature, and is easily executed and understood. One reasonable way to characterize our approach is to describe our target string of nucleotides *not* as what is required to create a given new gene or function, but rather our target string is what is lacking, as needed to complete a new gene or function that is already serendipitously near completion and only lacks the specified string of mutations. With this definition of our target string we can reasonably require that every nucleotide in our initial string be mutated, that all point mutations be neutral until the string is complete, and every nucleotide must eventually match the corresponding target nucleotide before there is any reproductive benefit for the carrier individual.

## Methods

When we consider the nature of the problem stated above, it might seem that analytical approximation would be sufficient for estimating the waiting time required for generating and fixing any specific target string. It is true that analytical approximation can be helpful in making very crude estimates of waiting times, but it requires gross over-simplification of the problem and ignores many important biological factors that can profoundly affect time to fixation. As we will show, comprehensive numerical simulation requires fewer simplifying assumptions, and therefore provides estimates of waiting times that are more reliable and more informative compared to analytical approximations.

To do these simulation experiments, we employed the numerical simulation program *Mendel’s Accountant* (Mendel version 2.4.2, now being released as 2.5). This new version was specifically designed to enable these types of waiting time experiments, and is freely available for download at SourceForge [27]. The current downloadable Mendel version is for Mac computers only, and is not compatible with all Mac operating systems. Alternative versions for use with other computer systems will be available at http://www.mendelsaccountant.info/ [28]. Detailed instructions on how to use Mendel to execute waiting time experiments are also available [28].

Mendel is arguably the first comprehensive, biologically realistic simulator of the mutation/selection process [29–36]. Mendel allows the user to specify all of the major biological variables that affect selection efficiency. Mendel faithfully models our present understanding of the neo-Darwinian mutation/selection process and maintains biological realism at a level that has not been previously possible. Mendel is user-friendly and enables the user to adjust over 25 relevant parameters. Mendel simulates a virtual population, creates new mutations and applies them to individuals, followed by computation of individual fitness, probability selection based on each individual’s fitness, random mating, and then progeny production. This process is repeated for a designated number of generations, with every mutation being tracked. Output is automatically tabulated and plotted, revealing the overall outcome of the mutation/selection process.

Our simulations track the fixation process through three phases. Stage 1 is the waiting time to first instance of the target string within the population. This first instance of the string is almost always a dead-end, being lost from the population shortly after it arises, due to genetic drift. The target string, even though it is beneficial and is subject to amplification via natural selection, must generally arise many times before it will “catch hold” within the population. Stage 2 is the waiting time to that special instance (the *effective instance*) of the string in the population that escapes extinction due to drift, and so will eventually go to fixation. Stage 3 is the waiting time for the effective instance to be amplified by selection to the point of fixation. Comprehensive numerical simulation is very valuable for fully understanding the waiting time associated with each stage of the generation/fixation process, because all three stages are strongly affected by interacting biological parameters.

Unless otherwise specified, we used the default settings of Mendel’s Accountant. Fertility rate was set at the default setting, such that 50 % of all progeny were selectively eliminated every generation – reflecting very intense selection. The human mutation rate per nucleotide per generation is known to be slightly higher than 10^−8^ [10–12]. The mutation rate we used (per string) was calculated by multiplying 10^−8^ times the number of nucleotides in the string, times two (because the string is diploid). As needed, the mutation rate was enhanced, as described in the results section.

Mendel was specially modified for this research, allowing us to: 1) define a starting nucleotide string of a given length which resides at a specific location within the virtual genome; 2) define a corresponding target nucleotide string of the same length which may or may not share nucleotides with the starting sequence; 3) cause the initial string to undergo random single nucleotide substitutions (point mutations) at a specified rate; and 4) whenever the target string arises, Mendel confers a reproductive benefit to any individual carrying the target string. In other words, whenever random mutations give rise to the target string, it results in a fitness benefit for all carrier individuals such that those individuals have an enhanced probability of reproduction. This initiates the selective amplification process.

Mendel was modified so that once a string mutates into the target string, that string would be protected from further mutations. Otherwise, when mutation rates are significantly enhanced to allow faster runs, the elevated mutation rates could result in significant back-mutation pressure. This artificial constraint was generous in terms of accelerating the time to fixation, because it prevented any mutational degradation of the target string after it arises.

When natural selection successfully amplified the target string to the point of fixation (i.e., when the allele frequency reached 99–100 %), the experiment was automatically halted and summary statistics were calculated and plotted. The summary statistics include: a) waiting time to first instance of the target string; b) waiting time to the effective instance which will eventually “take hold” and go to fixation; c) duration of the selective amplification phase; d) total fixation time, and e) total number of independent instances of the target string that arose before the final fixation event.

All experiments were replicated 25 times, to determine average waiting times and average number of instances required. The following biological variables were tested to see how they impacted waiting times: a) mutation rate; b) string length; c) degree of fitness benefit; and d) population size.

### Implementation details

The details of how Mendel’s Accountant operates are described in detail elsewhere [29]. The approach used here to model the realization of a target nucleotide sequence through mutation differs from the way in which Mendel normally handles mutations. Normally in Mendel mutations are given a unique identification number that encodes both their fitness effect value and their location in the genome. By contrast, for the cases described in this paper, it is the actual nucleotides themselves that are tracked. The treatment assumes there is but one chromosome and that the number of linkage blocks on that chromosome is equal to the user-specified target string length such that each linkage block carries a single nucleotide location. An array is used to store the values 0, 1, 2, or 3 - which represent the four nucleotides A, C, T, and G, respectively, at each linkage block/nucleotide location along the chromosome. At the beginning of a run, the nucleotide sequence on each chromosome of each individual in the population is set either to a randomly generated sequence or to a sequence specified by the user (such as AAAAA). New mutations that occasionally arise in an offspring correspond to a random change in one of the nucleotides along the single chromosome in the case of a haploid organism or along one of the pair of chromosomes in a diploid organism. Such a mutation overwrites the previous nucleotide associated with that specific site.

Each time a new mutation arises in an offspring, the entire string on the affected chromosome is checked against the target sequence. If the mutated string matches the target string, the value in a target-match array is changed from zero to a random value between one and a million. This nearly unique value in the target-match array allows Mendel to track each of the distinct instances in which the target sequence has arisen via mutation in the population during its history. Currently we inhibit back-mutation, such that if the target string has been matched, no further mutations on that chromosome are allowed. In the selection subroutine, each individual that has a non-zero value in the target-match array receives a fitness ‘bonus’ specified as a user-specified parameter. In the case of diploid organisms the fraction of the full bonus the individual receives depends on whether the target string is declared dominant or recessive and whether it is homozygous or heterozygous. During the selection phase, the individuals are sorted according to fitness, and the top NP individuals are selected to reproduce the next generation, where NP is the user-specified population size. NP is assumed constant in the cases we consider. The pseudo-code of the aforementioned approach is shown as follows for the diploid case, where NG is the user-specified maximum number of generations in the simulation:

- **initialization** : initialize every individual to the same random or user-specified string (e.g., AAAAA)
- **loop** over NG:
  - o**mating**: randomly mate half the population with members from the other half
    - ▪ **loop** over NP/2:
      - **offspring** subroutine:
        - o copy genetic makeup to offspring. Offspring receives half its genetic makeup from each of its parents by choosing randomly one haplotype from the mother and the other from the father.
        - o generate mutations. Based on the user-specified mutation rate, apply new random mutations in the linkage block/nucleotide array of the offspring genome. Test to determine if the string in mutated individual matches the target string. If so, load the target-match array with a random value between one and a million. Currently we prevent back-mutation.
    - ▪ **end** loop over NP/2
  - o**selection**:
    - ▪ compute fitness of each individual, adding an appropriate fraction of the user-specified “target match bonus” (e.g., 0.1) to each individual that has a target match for one or both chromosomes
    - ▪ impose selection based on phenotypic fitness to reduce the population size
  - o**termination criterion**: if portion of population with homozygous target match exceeds 99 %, then stop simulation and output results.
- **end** loop over NG

## Results

### Results of a series of simulation experiments that test the effect of mutation rate

Based upon our preliminary Mendel simulations and our preliminary mathematical approximations, it became obvious that we did not have the computational resources to do population simulations using the known mutation rate. The human mutation rate per nucleotide per generation is extremely low (roughly 10^−8^) [10–12]. If we used this actual mutation rate, string establishment would typically require many millions of simulation generations – ranging upward into the billions. In such cases we would be forced to simulate tens of thousands of generations of mating, mutation, and selection before even one random mutation arose within the string of interest anywhere in the whole population. To be able to do the many large simulations required in this study, given our available computational resources, we estimated that we needed to artificially increase the mutation rate by 10,000-fold. We hypothesized that we might safely increase the mutation rate by several orders of magnitude, observe waiting times, and then correct the waiting times by that factor.

We began by testing this working hypothesis. We chose parameter settings that were extremely generous, in terms of favoring the shortest possible waiting times. Our standard settings throughout this research (except where noted), were as follows: a) a fitness benefit of 0.1 (the completed target string confers a 10 % increase in total fitness); b) complete genetic dominance; and c) no other genetic variants arising or segregating within the genome. For this first series we used a string length of five nucleotides. We then did a series of simulations wherein we increased the natural mutation rate over a range of 2–5 orders of magnitude (i.e., by factors of 100; 1,000; 10,000; 100,000). In this way we greatly shortened both the biological and computational waiting times. We then corrected the waiting times by the same proportion by which the mutation rate was increased.

**Fig. 1:**
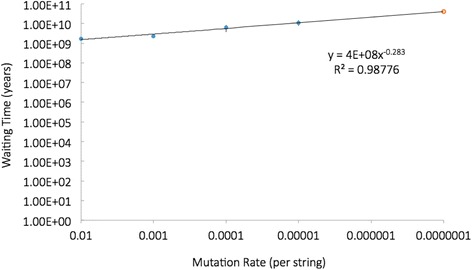
Corrected waiting time to fixation for a string of five nucleotides, across different mutation rates. Mutation rate is equal to mutations per diploid string per generation. Waiting times were adjusted proportional to the mutation rate enhancement (see text). Both scales are log10. (note: bottom scale moves from a highly unrealistic mutation rate (*far left*), to the actual mutation rate (far right). Extrapolation was necessary to determine the waiting time for the data point on the far right (*open red circle*) – which reflects the actual biological mutations rate. We anticipated that the line of best fit would be a horizontal line. The slope of the observed line of best fit is sloping upwards slightly – due to drift-induced string homogenization. The 95 % confidence intervals are shown, but are at best barely visible

We expected to see that waiting times, after correction, would be essentially the same using this procedure (the line in Fig. 1 would be horizontal). This expectation was largely realized, in that all adjusted waiting times were generally of the same order of magnitude (Table 1, Fig. 1). However, we were surprised to find that as we reduced our mutation rate (approaching the true mutation rate), waiting times systematically increased – even after application of the proportional correction factor (Table 1, Fig. 1). We examined our simulations more carefully, to understand why lower (more realistic) mutation rates disproportionately extended the waiting time to fixation. Interestingly, we found the problem was not computational, but indeed had a biological explanation. Lower mutation rates resulted in longer runs (requiring more generations per new mutation), and consequently almost all variant strings (alleles) were being eliminated by genetic drift. Drift was eliminating variants about as fast as new mutations were happening. This was resulting in near-zero genetic variance of the string within the population. We saw that a given random neutral string (allele) would drift to become the primary allele within the population, and then would drift out again and be replaced by another random allele. The effect of such strong homogenization of the string population via drift was similar to having a reduced effective population size, and this resulted in a significantly longer time to find the target sequence. When seeking a specific target string, it is optimal to have the population filled with the widest possible range of string variations. It is especially important to have intermediate strings within the population that are “within striking range” (nearly identical to the target - such near-miss strings can themselves be very rare, especially when strings are longer than three). Because genetic drift dramatically reduces the number of string variants within the population, it makes the essential “near-miss” strings even more rare than expected. Having almost no variation in the string population, and having almost no near-miss sequences, obviously results in longer waiting times for finding the target. Comprehensive numerical simulation revealed to us the key role of drift-induced string homogenization as a major limiting factor in finding the target sequence.

**Table 1:** Average corrected waiting times for a string of five nucleotides

Mutation rate per string per generation	Average number of instances before fixation	Average corrected waiting time to 1st instance (yrs)	Average corrected waiting time to effective instance (yrs)	Average duration of amplification (yrs)	Total average corrected waiting time (yrs)	95 % Confidence Interval for total waiting time (yrs)
0.01	15.6	1.05 × 10^9^	1.72 × 10^9^	1.80 × 10^4^	1.72 × 10^9^	2.46 × 10^8^
0.001	9.0	1.70 × 10^9^	2.31 × 10^9^	1.44 × 10^4^	2.31 × 10^9^	4.98 × 10^8^
0.0001	8.7	4.43 × 10^9^	6.10 × 10^9^	1.60 × 10^4^	6.10 × 10^9^	2.39 × 10^9^
0.00001	7.6	8.53 × 10^9^	1.11 × 10^10^	1.67 × 10^4^	1.11 × 10^10^	3.42 × 10^9^
*0.0000001* *biological rate*					*3.95* × *10* ^*10*^ *(extrapolated)*	

The correction factor was proportional to the mutation rate enhancement, but was not applied to duration of the amplification phase. Each data point is the mean of 25 replicates. Population size (10,000) and beneficial fitness effect (10 %) were held constant. Mutation rates and correction factors range over four orders of magnitude. The last row (in italics) reflects the true mutation rate, which requires computational resources not presently available. This last data point is derived by extrapolation using the equation based on the line of best fit in Fig. 1

Throughout the remainder of our study we enhanced the mutation rate 10,000-fold during the simulations, followed by a 10,000-fold correction factor of waiting times after the experiment. We did not attempt to adjust for drift-induced string homogenization via extrapolation (as discussed above). The extrapolated waiting time for the actual mutation rate was roughly one order of magnitude higher than our corrected waiting time when using our standard mutation rate of .001 (Table 1, Fig. 1). Because we used the more conservative method of simple proportional correction for mutation rate, all of our reported waiting times in this paper actually underestimate waiting time by roughly a factor of ten (Table 1).

A minor refinement in determining total fixation time involved applying the mutation rate correction factor to the time to first instance and also to the time to effective instance, but not applying the correction factor to the amplification interval that leads to final fixation. This last stage of the fixation process (the selective amplification phase) is not dependent on any further mutations and is independent of mutation rate. Had we failed to take this into account, we would have obtained waiting times slightly longer than what we report here.

### Results of a series of simulation experiments that test the effect of string length

We next tested the effect of string length on waiting time, using the same generous parameter settings that were described above (10 % beneficial fitness effect, full dominance, no other genetic variants in the genome). Because the actual mutation rate per nucleotide is so very low, we see that even changing a specific single nucleotide to a specific alternative (beneficial) nucleotide required a problematic waiting time (see Discussion). The average waiting time for the fixation of such a “string of one” was 1.53 million years (Table 2). For this single nucleotide substitution, the average time to first instance was 189,000 years, average time to the effective instance was 1.52 million years, and average duration of the selective amplification phase was 16,500 years.

**Table 2:** Corrected waiting times for single nucleotide substitutions, and strings of 2–8 nucleotides

String Length	Average number of instances before fixation	Average waiting time to 1st instance (yrs)	Average waiting time to effective instance (yrs)	Average duration of amplification stage (yrs)	Total average waiting time (yrs)	95 % confidence intervals for total average waiting time (yrs)
1	27.4	1.89 × 10^5^	1.52 × 10^6^	1.65 × 10^4^	1.53 × 10^6^	4.03 × 10^5^
2	15.0	2.79 × 10^7^	8.40 × 10^7^	1.72 × 10^4^	8.41 × 10^7^	2.01 × 10^7^
3	6.8	2.20 × 10^8^	3.76 × 10^8^	1.73 × 10^4^	3.76 × 10^8^	6.98 × 10^7^
4	12.6	8.30 × 10^8^	1.22 × 10^9^	1.93 × 10^4^	1.22 × 10^9^	1.72 × 10^8^
5	9.0	1.70 × 10^9^	2.31 × 10^9^	1.44 × 10^4^	2.31 × 10^9^	4.98 × 10^8^
6	8.2	3.16 × 10^9^	4.24 × 10^9^	1.62 × 10^4^	4.24 × 10^9^	1.02 × 10^9^
7	6.4	5.52 × 10^9^	8.59 × 10^9^	1.71 × 10^4^	8.59 × 10^9^	2.16 × 10^9^
8	9.3	1.11 × 10^10^	1.85 × 10^10^	1.69 × 10^4^	1.85 × 10^10^	7.25 × 10^9^

Each value is the mean of 25 replicates. The population (10,000) and beneficial fitness effect (10 %) were held constant. The mutation rate was adjusted based on number of nucleotides in the string. For single point mutations (string length of one), numerous instances accumulated simultaneously in the population – such that many were superfluous to waiting time, resulting in extra instances arising prior to fixation

**Fig. 2:**
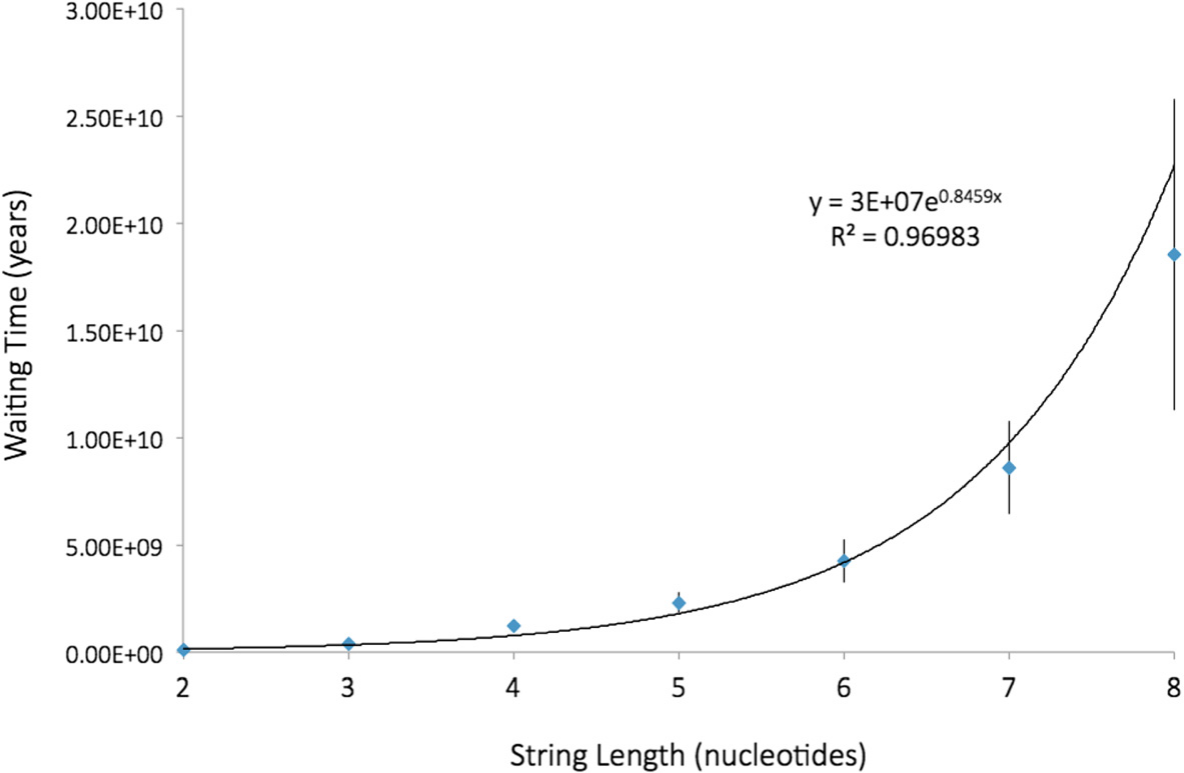
Corrected waiting time to fixation for different nucleotide string lengths. Each point represents the mean of 25 replicates. Population size (10,000), fitness effect for the target string (10 %), and the mutation rate per nucleotide were all held constant (mutation rate per string increased proportional to string length). The 95 % confidence intervals are shown, but are barely visible in the shorter strings

Table 2 also shows the waiting times for creating and fixing genuine strings of 2–8 nucleotides. This data is also plotted in Fig. 2. Each additional nucleotide that was added to the target string substantially increased waiting time. Figure 2 shows that as string length increased linearly, the increase in waiting time was of an exponential nature. When there were as many as six nucleotides in the string, the average waiting time (4.24 billion years) approached the estimated age of the earth. When there were eight nucleotides in the string, the average waiting time (18.5 billion years), exceeded the estimated age of the universe.

### Results of a series of simulation experiments that test the effect of fitness benefit

**Fig. 3:**
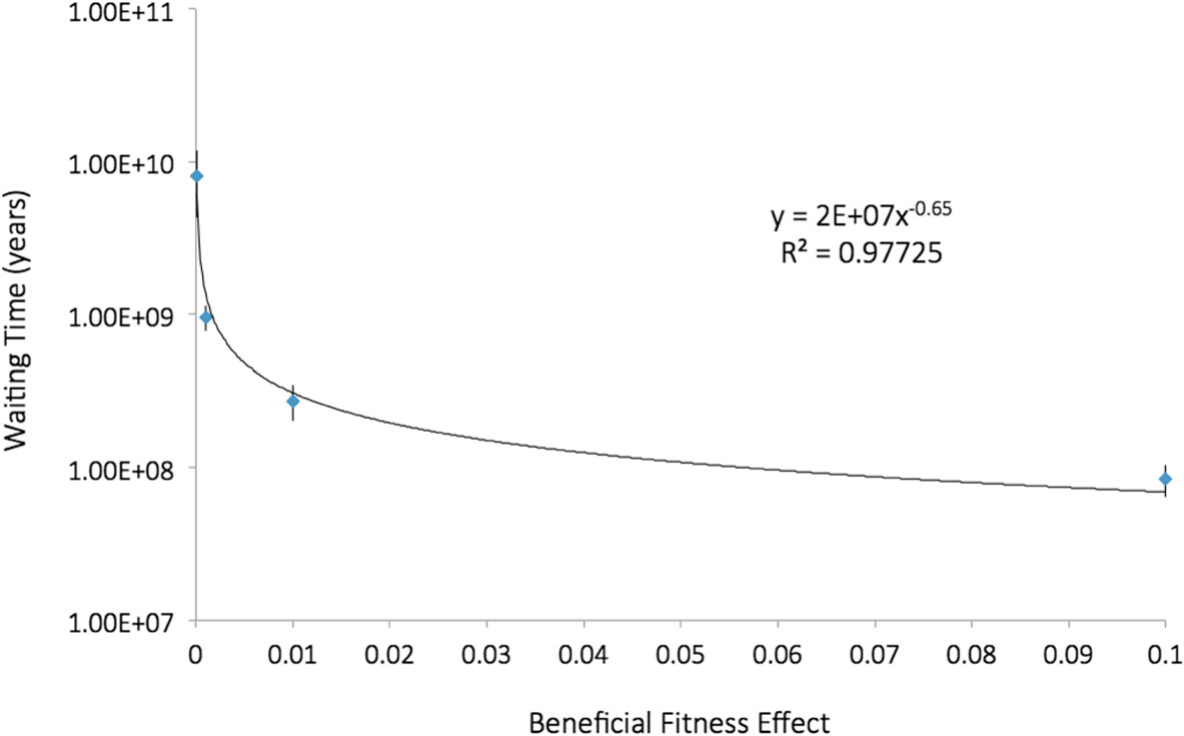
Corrected waiting times for a string of two, depending on strength of beneficial effect. Beneficial fitness effects were 0.1, 0.01, 0.001, 0.0001. Each point represents the mean of 25 replicates. The population size (10,000) and mutation rate (0.0004) were held constant. The 95 % confidence intervals are shown

In a genome with over three billion nucleotides, a very short string of nucleotides cannot normally be expected to encode a very large increase in total fitness (such as a 10 % increase in total functionality of the organism). It is widely understood that most beneficial mutations should confer very modest increases in total fitness. Mutational fitness benefits are typically orders of magnitude less that 10 %. In this series of experiments, we included more realistic beneficial fitness effects for the completed string ranging from 10 % down to .01 % (i.e., fitness effects of 0.1; 0.01; 0.001; 0.0001). Using Mendel we simulated the shortest possible string (two nucleotides). We did this because when combined with realistically modest beneficial effects, even a string length of two generally resulted in prohibitive average waiting times (100 million to over a billion years). See Table 3, and Fig. 3.

**Table 3:** Corrected waiting times for a string length of two, as beneficial fitness effect varies

Fitness increase	Average number of instances before fixation	Average waiting time to 1st instance (yrs)	Average waiting time to effective instance (yrs)	Average duration of amplification stage (yrs)	Total average waiting time (yrs)	95 % confidence intervals for total average waiting time (yrs)
0.0001	19,800	3.00 × 10^7^	8.10 × 10^9^	7.00 × 10^5^	8.10 × 10^9^	3.78 × 10^9^
0.001	3,079	3.05 × 10^7^	9.59 × 10^8^	3.35 × 10^5^	9.59 × 10^8^	1.78 × 10^8^
0.01	221	2.77 × 10^7^	2.73 × 10^8^	7.35 × 10^4^	2.73 × 10^8^	7.10 × 10^7^
0.1	15	2.79 × 10^7^	8.40 × 10^7^	1.72 × 10^4^	8.41 × 10^7^	2.01 × 10^7^

Each value represents the mean of 25 replicates. The population size (10,000) and mutation rate were held constant. A beneficial fitness effect of 0.1 is the same as a 10 % increase in total functionality

### Results of a series of simulation experiments that test the effect of population size

**Fig. 4:**
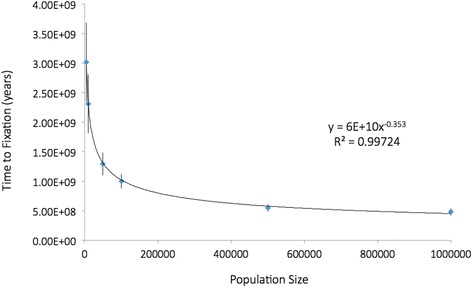
Corrected waiting time for fixation of a string of five, depending on population size. Each point represents the mean of 25 replicates. The mutation rate (0.001) and fitness effect (10 %) were held constant. Waiting times for population size of 1000 are shown in Table 4, but are off scale for this plot. The 95 % confidence intervals are shown

Larger population sizes can significantly reduce waiting time to first instance of the target string, and hence can reduce total waiting time. The effect of increasing population size was tested for a string of five, assuming the same generous biological parameter settings (10 % fitness benefit, full dominance, no other genetic variants in genome). Results of this series of experiments are summarized in Table 4 and Fig. 4. Increasing population size from 1,000 to 10,000 resulted in a 5.5-fold decrease in average waiting time. Further increase from 10,000 to 100,000 resulted in a further 2.3-fold decrease in average waiting time. Another 10-fold increase from 100,000 to 1,000,000 individuals resulted in only a two-fold reduction in average waiting time. It is very clear that larger population size results in a rapidly diminishing return in terms of shorter waiting time (Fig. 4). Furthermore, even with a population size of one million, waiting time was 500 million years – which is still extremely prohibitive. This amount of time approximates the estimated time required for the evolution of worm-like creatures into people. Our computational resources did not allow us to simulate populations larger than one million, but Table 4 includes extrapolations of population size to ten million (202 million years) and one billion (39.9 million years). It is very clear that increasing the population size leads to rapidly diminishing returns in terms of shortening waiting time.

**Table 4:** Corrected waiting times for a string of five, depending on population size

Population size	Average number of instances before fixation	Average waiting time to 1st instance (yrs)	Average waiting time to effective instance (yrs)	Average duration of amplification stage (yrs)	Total average waiting time (yrs)	95 % confidence intervals for total average waiting time (yrs)
1000	11.2	6.76 × 10^9^	1.28 × 10^10^	1.01 × 10^4^	1.28 × 10^10^	5.61 × 10^9^
5000	9.2	2.31 × 10^9^	3.01 × 10^9^	1.40 × 10^4^	3.01 × 10^9^	6.70 × 10^8^
10000	9.0	1.70 × 10^9^	2.31 × 10^9^	1.44 × 10^4^	2.31 × 10^9^	4.98 × 10^8^
50000	10.5	8.54 × 10^8^	1.29 × 10^9^	2.19 × 10^4^	1.29 × 10^9^	1.92 × 10^8^
100000	12.0	6.86 × 10^8^	9.99 × 10^8^	2.34 × 10^4^	9.99 × 10^8^	1.18 × 10^8^
500000	11.5	3.97 × 10^8^	5.50 × 10^8^	2.53 × 10^4^	5.50 × 10^8^	6.15 × 10^7^
1 million	10.0	3.30 × 10^8^	4.82 × 10^8^	2.57 × 10^4^	4.82 × 10^8^	6.23 × 10^7^
*10 million*					*2.02* × *10* ^*8*^ *(extrapolated)*	
*1 billion*					*3.99* × *10* ^*7*^ *(extrapolated)*	

Each value represents the mean of 25 replicates. The mutation rate (0.001) and beneficial fitness effect (10 %) were held constant. The values in the last two rows (in italics) are extrapolated using the equation based on the line of best fit in Fig. 4

A key reason why larger population sizes do not more completely resolve the waiting time problem for nucleotide strings is that increasing population size does not increase *mutation density*. A large enough population ensures that any single point mutation will arise somewhere in the population every generation. But at that point, specific sets of mutations still remain extremely rare. A bigger population increases the number of mutations arising per generation, but does not increase the number of mutations per short DNA strand (mutation density). To create a complete set of linked mutations requires many mutations arising on the same short stretch of a given DNA molecule. To generate a string of five nucleotides, minimally five specific mutations are needed on the same short stretch of that specific DNA molecule (but obviously not in the same generation). In reality, the average number of mutations that must happen on that same short stretch of DNA of length ‘n’ must approach 4^n^ before we are likely to see the first instance of the target arise anywhere in the population. So for a string of five, this means it is necessary to wait until almost 1,024 mutations have occurred within the same short stretch of a given DNA molecule. The need to have so many mutations arise in the same small linkage group can be termed “the mutation density problem”. Larger population sizes cannot resolve the mutation density problem. In a very large population it can take almost no time to get any particular point mutation, but regardless of population size it still takes a very long time to create specific strings of more than three nucleotides. Analytical approximation might not have revealed this important insight.

## Discussion

We have focused on the waiting time problem as it would apply to an evolving hominin population of 10,000-100,000. It is within this type of population where the waiting time problem is most acute, due to long generation time and limited population size. This type of mammalian population is small enough so that it is possible to use comprehensive numerical simulation experiments – simulating all the essential variables simultaneously and producing results that are biologically realistic. While we believe our simulation results are broadly informative, we do not assume our results are directly transferable to microbial organisms. Simulating mega-populations using comprehensive numerical simulators such as Mendel will be possible only when adequate computing resources become available. Therefore we limit all our discussion to a relatively small hominin population.

Our numerical simulations consistently reveal that in such a population the waiting time problem is profound. Even waiting for the fixation of a single point mutation that converts a specific nucleotide within the genome into a specific alternative nucleotide is problematic. The waiting time for the establishment of such a simple event requires on average over 1.5 million years (Table 2). This is a very long time to wait for such a tiny genetic modification of a pre-human genome. It causes us to ask; “Is such a long waiting time credible?”

The ballpark figure for establishing a specific point mutation is easily validated using very straightforward analytical approximation. The human mutation rate is approximately 10^−8^ per nucleotide per generation (see [10–12]). This means that the waiting time for a specific nucleotide within single chromosomal lineage would be 100 million generations. In a diploid population of 10,000 there are 20,000 copies of each nucleotide, so in such a population the waiting time to first mutation would be 20,000 - fold less (5,000 generations). But to have that specific nucleotide mutate into a specific alternative nucleotide (there are three alternatives), takes three times more time – which is 15 thousand generations. But even if the mutation is highly beneficial, on average it has to happen very roughly ten times (given the 10 % fitness benefit), before it “catches hold”. This yields 150,000 generations. Assuming a generation time of 20 years, the waiting time is about three million years. So analytical approximation yields a waiting time of very roughly 3 million years for the establishment of a specific point mutation, given a very strong fitness effect of 10 %. Our numerical simulations yielded a comparable waiting time (1.53 million years), which is in the same ballpark (actually being slightly shorter). This makes it clear that our simulation methodology is not yielding unreasonably long waiting times and is in fact conservative. The problem is not in our numerical simulations - rather it is in the biology. As a general rule, small mammalian populations must wait a very long time for a specific point mutation to arise and be fixed.

There are two primary reasons for such a long waiting time, even for the fixation of a single specific substitution: 1) the mutation rate per nucleotide site is so extremely low; and 2) a particular mutation needs to occur many times before it can “catch hold” in the population. Any analysis that equates waiting time to first instance with total waiting time will be grossly in error. Because we used a very large fitness benefit of 10 % for most of the simulations in this study, we minimized the time required for the mutation to catch hold. Therefore, our reported waiting times are very conservative. Indeed, we have generally coupled our exaggerated fitness benefits with full dominance and zero selection interference (we ignore all the other parts of the genome that would be mutating and segregating, which would otherwise result in selection interference and longer waiting times). Therefore, our results generally represent best-case scenarios in terms of minimizing waiting time. When we use more realistic parameter settings for our simulations, we consistently get much longer waiting times.

When we have reduced the single point mutation’s fitness benefit to a more realistic level of 1 %, waiting time increases ten fold (15.9 million years, rather than 1.5 million years). Given an even more reasonable fitness benefit of 0.1 %, average waiting time was 145 million years. So allowing for a more realistic range of fitness effects, we should more accurately say that even given very substantial fitness effects, the waiting time for a specific point mutation ranges between 1.5 and 15.9 million years. This is consistent with comments by Durrett and Schmidt [16]. They only calculated waiting time to first instance, but at the end of their paper acknowledged that with a 1 % beneficial effect their waiting time to fixation would have been about 100 times longer – due to the need to wait for the effective instance. The need to wait 1.5 – 15.9 million years for the fixation of a particular point mutation is very sobering, since it is estimated that mankind evolved from a chimp-like creature in just 6 million years.

While total waiting time for a particular point mutation to arise and be fixed is surprisingly long in this type of population, the waiting time for any particular string of mutations is vastly longer. Waiting times increase dramatically as we increase the string length (Fig. 2). As shown in Table 2, if an eight-nucleotide string is required, waiting time exceeds the estimated age of the universe.

The severity of the waiting time problem as it applies to specific sets of mutations is due to four levels of constraint. The first level of constraint is that we still have the same low mutation rate per site as with a point mutation, but we need multiple point mutations to arise on the same short strand of DNA, which is very difficult and requires a certain *mutation density* (which larger population size fails to provide). The second level of constraint is that the average number of mutations that must arise on each short strand of DNA increases more or less exponentially with string length. While for a single point mutation there are three possible mutations (two of which are wrong), for a specific set of ‘n’ linked nucleotide positions there are 4^n^ possible strings (all of which are wrong - except one). The third level of constraint is that it is the *entire completed string* that must arise afresh, many different times, to overcome the problem of early extinction due to drift. The fourth level of restraint is that while a population is waiting (through deep time) for the correct string to arise, genetic drift is systematically eliminating almost all the string variants – including most of the necessary intermediate strings between the starting string and the target string. Given a modest population size and such a low mutation rate, genetic drift ensures that there will be only a few string variants within the population in any generation. So almost all mutations must arise within the random string that is currently dominant in the population. Almost all of the time there will be zero or essentially zero strings anywhere in the population that are even close to the target string.

Given optimal settings, what is the longest nucleotide string that can arise within a reasonable waiting time within a hominin population of 10,000? Arguably, the waiting time for the fixation of a “string-of-one” is by itself problematic (Table 2). Waiting a minimum of 1.5 million years (realistically, much longer), for a single point mutation is not timely adaptation in the face of any type of pressing evolutionary challenge. This is especially problematic when we consider that it is estimated that it only took six million years for the chimp and human genomes to diverge by over 5 % [1]. This represents at least 75 million nucleotide changes in the human lineage, many of which must encode new information.

While fixing one point mutation is problematic, our simulations show that the fixation of two co-dependent mutations is extremely problematic – requiring at least 84 million years (Table 2). This is ten-fold longer than the estimated time required for ape-to-man evolution. In this light, we suggest that a string of two specific mutations is a reasonable upper limit, in terms of the longest string length that is likely to evolve within a hominin population (at least in a way that is either timely or meaningful). Certainly the creation and fixation of a string of three (requiring at least 380 million years) would be extremely untimely (and trivial in effect), in terms of the evolution of modern man.

It is widely thought that a larger population size can eliminate the waiting time problem. If that were true, then the waiting time problem would only be meaningful within small populations. While our simulations show that larger populations do help reduce waiting time, we see that the benefit of larger population size produces rapidly diminishing returns (Table 4 and Fig. 4). When we increase the hominin population from 10,000 to 1 million (our current upper limit for these types of experiments), the waiting time for creating a string of five is only reduced from two billion to 482 million years. When we extrapolate our data to a population size of ten million we still get a waiting time of 202 million years. Even when we extrapolate to a population size of one billion we still have a waiting time of 40 million years. This is consistent with Fig. 3 of Lynch [15], which for a string of just two specific mutations (when *n* = 2), suggests extremely long waiting times in smaller populations, and suggests significant waiting times even in a population of 1 billion. As mentioned in our results section, it is true that a larger population size will always result in more mutations in less time, but it does not result in higher mutation-densities (more mutations arising in the same small linkage block of DNA), which is a critical factor limiting formation of specific nucleotide strings.

The only way around the profound waiting time problem within a hominin-type population is to invoke special, atypical circumstances. Different authors have invoked a variety of special circumstances to reduce waiting times. These special circumstances include: a) assuming fixation of the first instance of a string [16]; b) special strings with reduced context-dependence such as a protein binding-site [16, 17]; c) strings that are largely already in place, and only require one or two new mutations [15–17]; d) incomplete strings that are still considered beneficial and can be rewarded with a significant fitness benefit [15–17]; and e) extremely large population sizes [17]. These special circumstances can sometimes be honestly invoked to enable instances with greatly reduced waiting times, but they are not generically applicable, and so cannot be used as a general resolution to the waiting time problem. The generic waiting time problem remains unresolved.

The results of other researchers [20–24] are generally consistent with our own results. Interestingly, even the previous studies that have strongly argued against the waiting time problem [15–17] still indicate that for a hominin population, the fixation of two co-dependent mutations is extremely problematic. For example, in the analysis of Durrett and Schmidt [16], they studied the waiting time to first appearance (first instance) of various string types within a hominin-type population (a population essentially identical to our own simulated population and with exactly the same mutation rate). However, a specific formulation of the problem was chosen, designed for the special case of a protein-binding (regulatory) site. Several special cases were examined involving either reduced context constraint (many possible genomic sites), or reduced specificity restraint (incomplete strings are beneficial and selectable), or cases where the target string was already nearly complete (lacking only 1–2 nucleotide changes). So those results are only marginally comparable to the third column in all our tables (our time to first instance). In all these special cases, one would naturally predict significantly shorter waiting times than we report here. Yet for a string of 8, when a perfect match was required, they still calculated a waiting time to first instance of 650 million years. For a beneficial effect of 1 % they estimate that the time to the *effective instance* (followed by final fixation), would be about 100-fold higher (this would be about 65 billion years). Their results, when adjusted as they prescribe, make our own findings for a string of 8 seem quite modest (just 18.5 billion years). The primary reason our waiting time was less than their corrected waiting time was apparently because we used an over-generous fitness benefit 10 times stronger than what they were assuming.

In a second paper by Durrett and Schmidt [17], they examined a more limited problem – how long does it take to create two co-dependent mutations within a hominin population. Again, their formulation was designed for a special circumstance – switching a protein-binding site to a relatively non-specific alternative location. Yet their calculations indicated the average waiting time for establishment of these two mutations was still 216 million years (their simulations suggested a somewhat shorter time – 162 million years). Their waiting times again appear to be substantially longer than our own average waiting time for two co-dependent mutations (84 million years – Table 2). This again appears to be primarily because we used a ten-fold stronger fitness benefit. Their data is in good agreement with our own waiting time for two co-dependent mutations when we reduced our fitness benefit to a more reasonable 1 %. We then observed a waiting time of 270 million years (Table 3), which is in the same ballpark as their findings.

A paper by Lynch [15] suggests that the waiting time problem is not particularly serious – at least not for microbial populations. Yet that same analysis indicates a very significant waiting time problem for smaller populations. Figure 3 of that paper plots the waiting times for “neofunctionalization” (creation of a new function) after a gene duplication. When two mutations were required (needing to arise at two specific locations, such that *n* = 2), and when population size was 10,000, the waiting times goes off scale – exceeding 1 billion generations (which for a hominin population would be 20 billion years). Even with a population size of 10 million, this same plot seems to suggest that when *n* = 2 the waiting time would be prohibitively long for a model Hominin population - in the range of 10–100 million generations (i.e., 0.2 to 2 billion years). That paper’s formulation of the problem which is closest to our own (where fixation of two specific mutations are required within a population of 10,000) seems to suggest waiting times much longer than what we see in the current study. This again appears to largely be due to our use of such a strong fitness benefit - reflecting our decision to primarily model best-case scenarios.

The most recent attempt to resolve the waiting time problem is the paper by Lynch and Abegg [25]. The findings of that paper have been challenged by Axe [21]. The paper by Lynch and Abegg begins by acknowledging that the waiting time problem should be of great interest to the evolutionary community (“A central problem in evolutionary theory concerns the mechanisms by which adaptations requiring multiple mutations emerge in natural populations.”) These authors then suggest they have largely resolved that problem. However, that paper again suggests that for a hominin-type population, waiting times are much longer than we report in this paper. Figure 1 in that paper suggests that to establish two specific co-dependent mutations in a population of 10,000 (given that the first mutation to arise is neutral) requires roughly 10–100 million generations. In a hominin population this would be roughly 0.2 to 2 billion years – just to fix two specific mutations. Figure 1 in that paper also suggests that for the same population of 10,000, when the intermediate mutation has a deleterious effect of 1 %, the waiting time is nearly 100 billion generations (2 trillion years).

Lynch and Abegg [25] go on to invoke 3 special atypical circumstances (such as hyper-mutation) that might reduce waiting times, none of which would be generally applicable in our case (i.e., a hominin population of 10,000). Even when these special circumstances were assumed, prohibitively long waiting times were indicated for a hominin-type population of 10,000 (see Fig. 3 in that paper), consistently exceeding the waiting times we report here.

Lastly, Lynch and Abegg [25] also analyzed the waiting time required for strings longer than 2 nucleotides. They argue that beyond two mutations, longer string length has only a marginal effect on waiting time, especially in small populations. This conclusion is very counterintuitive and is strongly contradicted by our own findings (see our Fig. 2). Similarly, the findings of Durrett and Schmidt [16], and Axe [21], seem to directly contradict that claim. Even if the claim by Lynch and Abegg regarding string length were valid, all of their waiting times for strings longer than two were extremely prohibitive for a population of 10,000 (see Fig. 4 of that paper – also note, that plot employed a heightened fitness benefit of 2 %).

In light of all this, it is clear that there are multiple lines of evidence that support our findings. Firstly, our own mathematical approximations make us very confident that our simulations are yielding waiting times that are in the right ballpark (see above). Secondly, numerous other researchers have come to similar conclusions [13, 14, 20, 21, 23, 24, 26]. Lastly, the long waiting times we report here are even supported indirectly by the papers that have argued against a serious waiting time problem [15–17, 25]. When examined carefully, even those papers indicate that for a hominin-type population, waiting times are as long or even longer than we report here. Therefore, the theoretical evidence is very strong that there is a significant waiting time problem in any small hominin-type population, and this problem does not necessarily go away as the population gets larger.

When we began this research, our preliminary calculations and simulation experiments revealed to us that our observed waiting times were going to be extremely long. For this reason we tried to be conservative in terms of all our parameters. Firstly, we chose to use proportional correction of our elevated mutation rates, rather than correcting for the observed longer waiting times associated with drift-induced string homogenization. This would have yielded 10-fold longer waiting times (Table 1, Fig. 1). Secondly, we chose to use an extremely high fitness benefit of 10 %, rather than more realistic fitness effects – which would have yielded 10–100 fold longer waiting times (Table 3). Thirdly, we chose to use full dominance, rather than partial dominance – which would have yielded several-fold longer waiting times (data not shown). Fourthly, we disregarded all genetic variants within all other parts of the genome – which would have resulted in significant selection interference and much longer waiting times. Competing beneficial mutations [32] and deleterious mutations [37] both cause serious selection interference because simultaneous selection at countless other sites in the genome confounds selection for the target string. Ignoring all the other segregating sites in the genome greatly simplifies the waiting time problem, and more to the point, current computation capabilities are simply not able to track all mutations arising within the genome through such deep time. However, based upon previous studies [7, 32, 36], we know that selection interference at the genomic level can be severely limiting, and should greatly increase waiting times. For all these reasons, we view the analyses reported here as being a series of best-case scenarios that yield waiting times which are very conservative.

Which method of analyzing the waiting time problem is better - analytical approximation or comprehensive numerical simulation? We conclude that both methods can, and in this case do, yield very similar results. Regarding the subject of this research, both methods clearly show prohibitively long waiting times for establishing even the shortest nucleotide strings within a model hominin population of 10,000–100,000 individuals.

Comprehensive numerical simulation is a new research and teaching tool, and is useful for enhancing our understanding of population dynamics. This tool can be used to study population scenarios that might be too specific or too complex for the tradition methodology of employing analytical calculations (which require, by necessity, many simplifying assumptions). In terms of increasing clarity, comprehensive numerical simulation allows us to directly observe the detailed unfolding of the mechanistic process of mutation/selection. In this way comprehensive numerical simulation can counterbalance the very high degree of abstraction inherent in attempting to reduce complex population dynamics to simple mathematical formulas.

Comprehensive numerical simulation entails genuine empirical experimentation. Mendel experiments produce outcomes that are neither programmed nor formulated, but are truly emergent in nature – being solely dependent on the interaction of the numerous biological factors. This can produce results that are both unanticipated and instructive. Comprehensive numerical simulation has the potential to bring us greater clarity of understanding of complex population dynamics, and may even prompt the re-evaluation of some long-held prior assumptions. For these reasons, we believe comprehensive numerical simulation can serve to expand upon and complement the traditional analytical approach. These two methods can be used to enhance each other, providing independent testing, correction, validation, and refinement.

## Final considerations

### Alternative pathways can help reduce waiting time

It has been pointed out that alternative mutations can accomplish largely equivalent phenotypic changes and adaptations (15, 25). This is certainly true, especially when reductive evolution is taking places (there are many ways to break a gene, but very few ways to improve it). But even when considering instances of constructive evolution, there should be alternative pathways to meet specific needs. The existence of such alternative pathways should certainly reduce waiting times. If there is a single alternate string of the same length as our target sequence, and this alternative string can enable the same function, this would reduce the waiting time roughly by half. If there were ten alternative strings that yield the same solution, this would reduce waiting time roughly ten-fold. However, the waiting time problem for a model hominin population is so dramatic that we cannot even begin to resolve the problem – not even when we invoke the special case of having many alternative strings that all meet the same need. For example, this study has shown that even when given optimal settings, the establishment of a specific string of 2 requires an average waiting time of 84 million years. If we reduce this waiting time ten-fold we still need 8.4 million years – which is extremely problematic given the ape-to-man timeframe of 6 million years. To completely dispel the waiting time problem, one would need to invoke the existence vast numbers of alternative strings (all of which would be functionally equivalent), for every evolutionary challenge. The number of potential solutions to a given problem has a great deal to do with the nature of the problem. If size reduction is what is required, there are clearly many ways to accomplish this. However if the ability to do calculus is what is required, the number of pathways may be very limited.

### Recombination may help reduce waiting time

This study, like most of the previous studies, only considers tightly linked point mutations and assumes no recombination within the string. This is a reasonable simplifying assumption but is not, of course, universally valid. However, the probability that a cross-over might occur between two specific adjacent nucleotides is extremely low, while a conversion event is only slightly more likely. Such rare recombination events should reduce the time to first instance of a target string, but only slightly and only when the two recombining sequences are genetically distinct from each other. Because genetic drift within a small hominin population is so strong over deep time, any two recombining strings will in most instances be identical. This makes the overall effect of recombination within a string exceedingly small.

Our general method of analysis is not inherently limited to strings consisting of immediately adjacent nucleotides. Eventually we will be able to apply our numerical simulations to any set of specific mutations located anywhere across the genome that mutually result in a fitness gain – with or without recombination. The Mendel program has not yet reached this level of development, but this is a potential topic for future research. Likewise, future research can examine mutations that are not entirely random, or that are not simple nucleotide substitutions.

### Beneficial mutational strings can simultaneously arise and can be fixed in other parts of the genome

It has been pointed out [15–17] that during the waiting time period for a functional string to be established at a given location, other beneficial mutational strings can be happening in other parts of the genome. This is certainly true, and for this reason we can expect some functional nucleotide strings to arise somewhere within the genome during the waiting time required to establish our special string of interest. However, those other strings are not likely to meet the same specific evolutionary need that our target string can meet. Evolution often needs a specific fix to a specific problem, and that fix must be timely in order to retain relevance.

Even if all of the ~20,000 genes in the hominin genome were already poised for a significant enhancement and all of them were waiting for their own specific string, each one of those potential enhancements would have its own severe waiting time problem. Certainly there could be simultaneous selection for several beneficial strings that might arise in different parts of the genome, but this would tend to only result in a very slow trickle of sporadic strings arising and being fixed, even within the whole genome, even in very deep time. If we had 20,000 independent strings-of-three under development, numerous strings might undergo staggered emergence over a period of several hundred million years - but we need to put this in perspective. In a genome of 3 billion nucleotides and in the context of extremely deep evolutionary time, the establishment of several thousand (or even several tens of thousands) of new nucleotides would reflect a statistically insignificant increase in information. Furthermore, this would be happening in the context of countless nearly-neutral deleterious mutations throughout the genome which would drift to fixation within the same deep time. Unless there was very strong purifying selection operating for all the nucleotides in the general region of the string, the context of the string would be erased long before the string itself actually arose. In this broader context, a net gain in total information/total functionality seems problematic.

### Not all strings may be context dependent

Normally, useful information is context-dependent. For example, short words like “no” or “yes” have no meaning apart from context. Within an instruction manual, it would be fairly easy to generate a word like “no” using a series of random word-processing errors. But it would be much harder to use this type of trial and error process to create such a word in a context that could make any sense. More difficult still, to create a biologically functional genetic element, random mutations must arise and be fixed to generate just the right word (i.e., “no”) – in just the right place (i.e., a specific codon) such that the whole genome is substantially improved and results in a new specified function that can significantly aid in an organism’s survival.

Genetic examples of context-specificity would be where a gene coding for a certain protein needs to substitute one amino acid for another, or where that same protein needs to be mutated so that it will have a particular new fold. There may be some actual biological examples where a very short text string might be beneficial regardless of context, but we are not aware of any such examples. Certainly the functionality of a short string apart from context would be rare, and does not reflect what is generally happening in the genome. For all these reasons we have modeled a context-dependent target string which must arise in a specific genomic location.

Behrens and Vingron [38] argue that in the special case of transcription binding factor sites, average waiting times can be dramatically reduced, because of the removal of various contextual constraints. For example, for a string of five, waiting time can be reduced almost 1000-fold, simply by assuming the target string can arise and be beneficial anywhere within the general vicinity of a 1000 bp promoter region. This special case may sometimes be true, but even given this very special circumstance, these authors demonstrate that for a hominin population the resulting waiting times are still very problematic (83M-338M years). Therefore, they invoke the extraordinary assumption that the target string can arise and be function anywhere within any of the 20,000 human promoters (reducing waiting time another 20,000-fold). This added assumption works mathematically, and can yield very short waiting times (about 7,000 years). However, biologically it is simply not reasonable to assume that a specific short string can be, by itself, beneficial almost anywhere in the genome, with minimal contextual constraint. We must not lose track of the real issue, which involves the average time required to *fix a specific set of linked mutations in a given population in order to establish a specific alteration in the genome*, such that *a specific new function is created that meets a specific evolutionary challenge*. 

### The string does not need to be complete before selection begins to operate

The concept of *functional threshold* is useful when considering the establishment of a beneficial nucleotide string. Any text string that specifies useful information has a functional threshold. The term “functional threshold” applies to the constructive process of building information. To encode a specific and meaningful new unit of information requires a minimal number of characters. This minimal number of characters is the functional threshold. In this research we have defined the length of the target string as the functional threshold for establishing a new function. In order for a string to be subject to positive selection it must reach this minimal functional threshold.

To illustrate this concept, suppose one is sending a text message to a friend, and it is accidently sent prematurely. The recipient will not be able to correctly understand the message unless the message had reached its functional threshold before being sent. Functional threshold is where a string of random characters has reached the point of completing some new, functional, meaningful unit of information. If someone is sending a text message that is meant to provide directions to their home, they cannot do it with any single character, or even any single word. They minimally need a string of characters specifying several words (or much more likely, a string of sentences). If someone were generating a series of random character-strings to their friend, hoping to eventually create directions that will guide them to a specific destination, it would take a very long time. It would take a long time even if the actual amount of required information was extremely small (i.e., a three-word sentence). This is also true when trying to create a specific functional string of just a few nucleotides.

In this paper, if the target sequence is five nucleotides long, we are not saying that five nucleotides are sufficient to create some new function, but rather we are saying that everything needed for the new function is serendipitously already in place, and all that is needed to reach the functional threshold is a set of five specific nucleotide changes. Only after these changes occur can natural selection begin to amplify the functional string and establish the new function in the population.

When text strings are required to convey meaningful information, it does not seem rational to hope that every word and every character will be independently beneficial. Biologically, larger strings might reasonably be built from smaller strings, but our intuitive understanding of information tells us that at some level short strings need to be the unit of selection if we are to build genomes and enable macroevolution. We cannot rationally expect that all biological information can arise exclusively by rewarding isolated point mutations – we need to generate biological text strings. So the question becomes; “How long does a nucleotide string need to be, to generate a selectable function?” Certainly the answer cannot be that every nucleotide is by itself beneficial and selectable. We suggest that selection for intact sets of nucleotides must routinely be invoked to enable the building of genomes.

We know that the standard definition of the linguistic functional threshold is a complete sentence. For example, there is a coherent unit of information within the sentence, “Modulate the expression of gene ABC.1234 to compensate for temperature” (70 characters, including spaces). Even though complete, this unit of information is not independently functional - it implies the need for an external modulating function, an external gene identification function, and an external temperature sensing function. In a practical sense we might wish to abbreviate or truncate this specification, for example it might be reduced to “Mod. A/1 for temp.” with just 18 characters/spaces (but really this just hides most of the essential information, which must then reside somewhere else). Taken to the extreme such abbreviation might be just a few characters long (“Mod-X”). But even “Mod-X” requires five characters. Whether we use the full sentence or use extreme abbreviation, we cannot reasonably expect that by pure luck every letter (starting with the first letter ‘M’) will consistently constitute a unit of information that is both independently functional and independently selectable. Regardless of where we draw the line of selectable functionality, we cannot realistically expect to be able to create vast networks of biological information by having natural selection act exclusively on each nucleotide independently.

Almost universally, we understand that functional *sets of characters* are needed to create any meaningful, new, prescriptive information. Arguably, biological information networks must contain vast numbers of functional strings with functional thresholds of two or more nucleotides. Many orphan genes encode novel proteins that have no known ancestral homolog. Such orphan genes seem to have functional thresholds of a thousand or more nucleotides. Yet in this paper we show that in a small mammalian population it is not generally feasible to even establish a string of five nucleotides, not even in a billion years. This is a very interesting theoretical dilemma.

## Conclusions

We have used comprehensive numerical simulations to show that in populations of modest size (such as a hominin population), there is a serious waiting time problem that can constrain macroevolution. Our studies show that in such a population there is a significant waiting time problem even in terms of waiting for a specific point mutation to arise and be fixed (minimally, about 1.5 million years). We show that the waiting time problem becomes very severe when more than one mutation is required to establish a new function. On a practical level, the waiting time problem greatly inhibits the establishment of any new function that requires any string or set of specific linked co-dependent mutations. We show that the waiting times problem becomes more extreme as string length increases, as fitness benefit decreases, and as population size decreases. In a population of 10,000 the establishment of a string of just two specific co-dependent mutations tends to be extremely problematic (conservatively requiring an average waiting time of at least 84 million years). For nucleotide strings of moderate length (eight or above), waiting times will typically exceed the estimated age of the universe – even when using highly favorable settings. Many levels of evidence support our conclusions, including the results of virtually all the other researchers who have looked at the waiting time problem in the context of establsihing specific sequences in specific genomic locations within a small hominin-type population. In small populations the waiting time problem appears to be profound, and deserves very careful examination. To the extent that waiting time is a serious problem for classic neo-Darwinian theory, it is only reasonable that we begin to examine alternative models [39, 40] regarding how biological information arises.

## References

[1] Britten RJ (2002). Divergence between samples of chimpanzee and human DNA sequence is 5 % counting indels. Proc Natl Acad Sci U S A..

[2] Haldane JBS (1957). The cost of natural selection. J Genetics..

[3] Kimura M (1968). Evolutionary rate at the molecular level. Nature..

[4] Bataillon T (2000). Estimation of spontaneous genome-wide mutation rate parameters: whither beneficial mutations?. Heredity..

[5] Bataillon T, Bailey SF. Effects of new mutations on fitness: insights from models and data. doi:10.1111/nyas.12460 Ann N Y Acad Sci. 2014;1320:76–92. 10.1111/nyas.12460 PMC4282485 24891070

[6] Elena SF, Ekunwe L, Hajela N, Oden SA, Lenski RE (1998). Distribution of fitness effects caused by random insertion mutations in Escherichia coli. Genetica.

[7] Gerrish PJ, Lenski RE (1998). The fate of competing beneficial mutations in an asexual population. Genetica.

[8] Montañez G, Marks R, Fernandez J, Sanford J, Marks RJ, Behe MJ, Dembski WA, Gordon BL, Sanford JC (2013). Multiple overlapping genetic codes profoundly reduce the probability of beneficial mutation. Biological Information – New Perspectives.

[9] Sanford. Genetic Entropy. 4th ed. FMS Publications; 2014.

[10] Lynch M (2010). Rate, molecular spectrum, and consequences of human mutation. Proc Natl Acad Sci U S A.

[11] Roach JC, Glusman G, Smit AFA, Huff CD, Hubley R, Shannon PT (2010). 2010. Analysis of genetic inheritance in a family quartet by whole-genome sequencing. Science.

[12] Campbell CD, Eichler EE (2013). Properties and rates of germline mutations in humans. Trends Genet..

[13] Behe MJ, Snoke DW (2004). Simulating evolution by gene duplication of protein features that require multiple amino acid residues. Protein Sci..

[14] Behe MJ, Behe MJ (2007). The mathematical limits of Darwinism. The Edge of Evolution.

[15] Lynch M (2005). Simple evolutionary pathways to complex proteins. Protein Sci..

[16] Durrett R, Schmidt D (2007). Waiting for regulatory sequences to appear. The Annals of Applied Probability.

[17] Durrett R, Schmidt D (2008). Waiting for two mutations: with applications to regulatory sequence evolution and the limits of Darwinian evolution. Genetics.

[18] Behe MJ (2009). Waiting longer for two mutations. Genetics..

[19] Durrett R, Schmidt D (2009). Reply to Michael Behe. Genetics..

[20] Axe DD (2010). The case against a Darwinian origin of protein folds. BIO-Complexity..

[21] Axe DD (2010). The limits of complex adaptation: an analysis based on a simple model of structured bacterial populations. BIO-Complexity..

[22] Gauger A, Ebnet S, Fahey PF, Seelke R (2010). Reductive Evolution Can Prevent Populations from Taking Simple Adaptive Paths to High Fitness. BIO-Complexity..

[23] Gauger A, Axe D (2011). The Evolutionary Accessibility of New Enzyme Functions: A Case Study from the Biotin Pathway. BIO-Complexity.

[24] Axe D, Gauger AK, Marks RJ, Behe MJ, Dembski WA, Gordon BL, Sanford JC (2013). Explaining metabolic innovation: neo-Darwinian versus design. Biological Information – New Perspectives.

[25] Lynch M, Abegg A (2010). The rate of establishment of complex adaptations. Mol Biol Evol.

[26] Reeves MA, Gauger AK, Axe DD (2014). Enzyme families-shared evolutionary history or shared design? A study of the GABA-aminotransferase family. BIO-Complexity..

[27] See SourceForge (http://sourceforge.net/projects/mendelsaccount/).

[28] See www.mendelsaccountant.info

[29] Sanford J, Baumgardner J, Brewer W, Gibson P, ReMine W. Mendel’s Accountant: a biologically realistic forward-time population genetics program. Scalable Computing: Practice and Experience. 2007;8(2):147–65. http://www.scpe.org/index.php/scpe/article/view/407.

[30] Sanford JC, Baumgardner J, Brewer W, Gibson P, ReMine W. Using computer simulation to understand mutation accumulation dynamics and genetic load. In: Shi Y, editor. ICCS 2007, Part II, LNCS 4488. Berlin, Heidelberg: Springer-Verlag; 2007. p. 386–92. http://bioinformatics.cau.edu.cn/lecture/chinaproof.pdf.

[31] Sanford J, Nelson C. (2012). The Next Step in Understanding Population Dynamics: Comprehensive Numerical Simulation, Studies in Population Genetics, in: M. Carmen Fusté (Ed.), ISBN: 978-953-51-0588-6, InTech, Available from: http://www.intechopen.com/books/studies-in-population-genetics/the-next-step-in-understanding-population-dynamics-comprehensive-numerical-simulation

[32] Sanford J, Baumgardner J, Brewer W, Marks RJ, Behe MJ, Dembski WA, Gordon BL, Sanford JC (2013). Selection Threshold Severely Constrains Capture of Beneficial Mutations. Biological Information – New Perspectives.

[33] Brewer W, Baumgardner J, Sanford J, Marks RJ, Behe MJ, Dembski WA, Gordon BL, Sanford JC (2013). Biological Information – New Perspectives. Using Numerical Simulation to Test the “Mutation-Count” Hypothesis.

[34] Brewer W, Smith F, Sanford J, Marks RJ, Behe MJ, Dembski WA, Gordon BL, Sanford JC (2013). Information loss: potential for accelerating natural genetic attenuation of RNA viruses. Biological Information – New Perspectives.

[35] Baumgardner J, Brewer W, Sanford J, Marks RJ, Behe MJ, Dembski WA, Gordon BL, Sanford JC (2013). Can Synergistic Epistasis Halt Mutation Accumulation? Results from Numerical Simulation. Biological Information – New Perspectives.

[36] Gibson P, Baumgardner J, Brewer W, Sanford J, Marks RJ, Behe MJ, Dembski WA, Gordon BL, Sanford JC (2013). Can Biological Information Be Sustained By Purifying Natural Selection?. Biological Information – New Perspectives.

[37] Eyre-Walker A, Keightley P (1999). High genomic deleterious mutation rates in Hominids. Nature..

[38] Behrens S, Vingron M Studying the evolution of promoter sequences: a waiting time problem. Journal of Computational Biology. 2010;17:1591-1606. 10.1089/cmb.2010.0084 PMC3119604 21128851

[39] Hughes AL. Evolution of adaptive phenotypic traits without positive Darwinian selection. Heredity. 2012;108:347–53. 10.1038/hdy.2011.97 PMC3313059 22045380

[40] Marks II RJ, Behe MJ, Dembski WA, Gordon BL, Sanford JC, editors. Biological Information – New Perspectives. London: World Scientific; 2013. p. 1–563.

